# Prolonged use of foot abduction brace reduces the rate of surgery in Ponseti-treated idiopathic club feet

**DOI:** 10.1007/s11832-015-0663-y

**Published:** 2015-06-20

**Authors:** L. Shabtai, E. Segev, A. Yavor, S. Wientroub, Y. Hemo

**Affiliations:** Department of Pediatric Orthopaedics, Dana Children’s Hospital, Tel Aviv Sourasky Medical Center and the Sackler Faculty of Medicine, Tel-Aviv University, 6 Weizman Street, 64239 Tel Aviv, Israel

**Keywords:** Club foot, Foot abduction brace, Ponseti, Delayed ossification, Cartilaginous anlagen, Ossific nuclei of tarsal bones

## Abstract

**Purpose:**

There is conflicting evidence related to factors affecting the rates of recurrence of idiopathic club feet using the Ponseti method. We attempt to evaluate the predictors of success and failure in our physiotherapy-led Ponseti club foot clinic.

**Methods:**

We evaluated 189 children with 279 club feet with a mean follow-up of 6.3 years for the following: Pirani score at presentation, number of casts for correction, indication for Achilles tenotomy, and the duration of foot abduction brace (FAB) use, in relation to outcome. Outcome measures were the need for additional surgery and functional scores. Based on the pattern and rate of ossification of the tarsal bones in idiopathic club foot, a much longer FAB weaning protocol was designed and practiced since 2000. The objective of this study was to answer the question of whether a prolonged period of FAB use reduces the need for surgery in Ponseti-treated idiopathic club foot.

**Results:**

Thirty-six feet (12.9 %) underwent additional surgery. The Pirani score and the number of cast changes had no influence on the rate of surgery. The duration of FAB use had a significant effect on the outcome, i.e., the rate of surgery and functional scoring. Operated children used the FAB for 28 months versus 33 months in the non-operated group (*p* < 0.05). Only a minor delay in the attainment of walking age was noted (average 15 months).

**Conclusions:**

The duration of FAB treatment was found to be the most influential on the functional results and on rate of surgery. Close follow-up and longer FAB weaning program reduced the rates of recurrence.

## Introduction

The initial treatment of idiopathic congenital club foot is non-operative, with manipulation and serial casting according to the Ponseti method, followed by foot abduction orthoses (foot abduction brace, FAB) [1].

Recent studies have shown good initial results when the Ponseti method was applied by trained clinical specialists in either a teaching hospital setting or in a developing country [2–4]. The Ponseti method was as effective when it was performed by a physiotherapist as by a surgeon. Good continuity of care is essential to the success of the Ponseti method [2–6]. There are different reports of success rates and recurrence rates published in the medical literature [7].

Our club foot clinic was initiated in 1984. Care is provided by a team that comprises both a physiotherapist and orthopedic surgeons. The Ponseti method was adopted in our club foot clinic in the year 2000, and, since then, all new babies with club foot have been treated according to this method. The idea behind our clinic is that one should maintain close contact with the family in order to achieve high compliance rates using the FAB and detect early recurrences, if any. Treatment of the child with club foot continues for a long period of time after the last cast change and families need to be reassured and made aware of the importance of maintaining the brace, even though the child’s foot seems corrected. Our FAB weaning protocol is different from other medical centers; it is more gradual and lasts longer, taking into account the late ossification process of the tarsal bone in club feet.

The aims of this study were to evaluate our results using a Ponseti modified protocol for over 10 years with our gradual FAB weaning program, compare our results to other centers, and identify predictors for success or failure.

## Materials and methods

This is a retrospective study of all children with Idiopathic club foot treated with the Ponseti method in our club foot clinic from 2000 to 2010. The minimum follow-up from the last cast change was 2 years. All children were treated from the first month of life. The exclusion criteria consisted of: (a) non-idiopathic or atypical club feet, and (b) club feet treated for more than 2 months in other centers. At each visit, the Pirani score for severity was recorded. Cast changes were done by two orthopedic surgeons familiar with the method.

The following parameters were studied:

- Pirani score at presentation
- Number of casts before tenotomy
- Rate of tenotomies
- Duration of treatment with foot abductions orthoses (FAB)
- Attainment of walking age.

### Outcome measures

The primary outcome assessed the need for surgery, including intra-articular surgery (posterior release, posteromediolateral release, etc.), as well as extra-articular surgery, such as tendo-Achilles lengthening (TAL), tibialis anterior tendon transfer (TATT), and Steindler plantar fasciotomy. The secondary outcome measures were the functional score according to Ezra et al. [8], which consisted of a score for range of motion (ROM), heel position, forefoot appearance, presence of supination or cavus with gait pattern, shoe type, and pain, as well as parents’ satisfaction. Each parameter has its own score. The final score summarizes the score of all parameters. A score of 150 reflects optimal functioning. The functional assessment was performed by the physiotherapist only.

### Tenotomies

We perform the Achilles tenotomy in the outpatient clinic with local anesthesia. Only in cases of repeated tenotomy or in older children do we prefer to use general anesthesia and to perform open tenotomy rather than the percutaneous approach. We believe that tenotomy is a mandatory part of correcting the foot and only in a very few cases do we choose not to perform Achilles tenotomy. Only in cases where the heel pad was not empty and there was more than 20° of dorsiflexion did we choose not to perform Achilles tenotomy.

### Dana physiotherapist-led club foot clinic

Our goal is to achieve accurate assessment of the response to treatment and have a close follow-up in order to enable early detection of non-compliance/recurrence of deformity that deserves special attention. In order to minimize the holes in the net, patients and parents will have to schedule multiple clinic visits with the orthopedic surgeon in charge and in-between appointments with the physiotherapist. The physiotherapist allows personal contact and an open-door walk-in clinic to facilitate optimal communication with the parents. The follow-up is done by the pediatric orthopedic surgeon and by the physiotherapist at every cast change, from the beginning until the tenotomy. Once an FAB is applied, the child will be examined after 2 weeks by the physiotherapist to make sure the brace is used properly, to monitor compliance, to reassure the parents, and to reevaluate the foot correction. The child will be further examined every 6 weeks by the physiotherapist, and every 3 months by the orthopedic surgeon. At every visit, the child’s general health will be recorded, together with developmental milestones. In case of deterioration or early recurrence, a reevaluation will be done by the team. From the age of about 1 year, the child will be seen by the surgeon once in 6 months and in between, every 3 months, by the physiotherapist. The number of visits with the therapist depends mainly on the compliance of the family and the development of the child. In addition, the physiotherapist has a continuous phone dialog with the parents, in between clinic visits.

### FAB Dana weaning protocol

Based on the pattern and timetable of the ossification of the tarsal bones in idiopathic club feet, a weaning protocol was designed and has been in use since the adoption of the Ponseti treatment method by our department.

Once correction is achieved, the parents are instructed to use the FAB for 23 h a day, up to the age of 9 months. At this age, the FAB will be removed for 3 h a day. From 11 to 14 months of age, the brace will be used for 18 h a day, and, then, up to 2 years of age, the child will wear the brace during sleep and nap time only (12 h + 2 h). The brace-free time is always divided into two periods during the first 2 years. For the third and fourth years of life, the brace will be used at night only.

### Statistical analysis

Data were analyzed by calculating means and percentages, using the *t*-test and the χ^2^ test. The χ^2^ test was used when possible, and Fisher’s exact test was used when the χ^2^ test was not appropriate. For calculating correlations between continuous variables, we used the Pearson correlation. A two-sided *p*-value of <0.05 was considered to be statistically significant. Statistical analysis was performed using SPSS version 20 (SPSS).

## Results

We found 189 children with 279 club feet treated in our clinic from 2000 to 2010 that fulfilled our inclusion criteria. Seventy-five percent (142) were boys and 25 % (47) were girls. There was bilateral involvement of 47.3 % (90 children), while 52.7 % (99 children) had unilateral involvement.

The mean follow-up duration was 6.3 years (range 2–11 years). The mean Pirani score at presentation was 4.4 (range 2.5–6). The mean number of casts needed for correction was 6.3 (range 4–12 casts). There was a correlation between the initial Pirani score and the number of casts needed to achieve correction (Pearson correlation 0.354, *p* < 0.001).

Achilles tenotomy was performed in 270 out of 279 feet (96.8 %). Two children (2 feet) required re-tenotomy for incomplete correction.

Twenty-seven children with 36 feet out of 279 feet (12.9 %) required additional surgery.

No correlation was found between bilateral involvement and the need for additional surgery (χ^2^ = 0.004, *p* = 0.953). The different types of surgical procedures performed are summarized in Table 1. Patients with additional surgery had a mean of 6.5 cast changes, compared to 6.3 cast changes in patients without additional surgery (*p* = 0.4). The mean Pirani score at presentation was 4.5 in the operated feet, compared to 4.4 in the non-operated feet (*p* = 0.317).

**Table 1:** Types of additional surgeries

Surgery type	Number of feet	Percentage
TATT/PR/PF	13	35
TATT/PR	12	32.4
TATT only	3	8
TATT/TAL	3	8
PLR	2	5.4
CR	2	5.4
PR	2	2.7
Total	37	100

*TATT* tibialis anterior tendon transfer, *PR* posterior release, *PF* plantar fasciotomy, *TAL* tendo-Achilles lengthening, *PLR* posterolateral release, *CR* circumferential release

We found a significant correlation between the duration of using the FAB and the need for surgery (Table 2). In 58 children (82 feet) that were treated with the brace for only 2 years, 23 feet underwent additional surgery (28 %). In 59 children (87 feet) that were treated with the brace for 2–3.5 years, 12 feet underwent additional surgery (13.7 %). Of 29 children (48 feet) that were treated with the brace for more than 3.5 years, only one child underwent additional surgery (2 %). The remaining 43 children (62 feet) still use the FAB. The mean duration of treatment with the FAB in the operated group was 28 months, compared to 33 months in the non-operated group (*p* = 0.03).

**Table 2:** Rate of surgery and functional score in relation to foot abduction brace (FAB) duration

Parameter	Number of feet (patients)	Pirani score before treatment	Average no. of casts before tenotomy	No. of tenotomies	Surgery^a^	Functional score (range)	Walking age months (range)
FAB = 2 years	82 (58)	4.2 ± 0.87 (2.5–6)	6.5 ± 1.35 (4–12)	81/82 (98 %)	23/82 (28 %)	120 ± 18.01 (78–150)	15 ± 2.23 (11–22)
FAB > 2, < 3.5 years	87 (59)	4 ± 0.79 (3–6)	6.3 ± 1.23 (4–10)	83/87 (95 %)	12/87 (13.7 %)	134.3 ± 16.5 (65–150)	14.9 ± 2.18 (10–20)
FAB > 3.5 years	48 (29)	4.6 ± 0.93 (3.5–6)	5.8 ± 1.22 (4–9)	46/48 (94 %)	1/48 (2 %)	135 ± 10.63 (98–150)	15.7 ± 2.58 (12–20)
Still in FAB	62 (43)	4.7 ± 1.14 (4–6)	5.9 ± 1.66 (4–10)	60/62 (96.7 %)	N/A	131.3 ± 10.4 (113–150)^b^	16 ± 2.53 (12–22)
Total	279 (189)	4.4 ± 0.91 (2.5–6)	6.3 ± 1.40 (4–12)	270/279 (96.8 %)	36/279 (12.9 %)	131 ± 14.85 (65–150)	15 ± 2.42 (10–22)

*N/A* not available

^a^Significance at *p* < 0.05

^b^At last visit

The mean functional score for all children included in this study was 131 points (range 65 to 150 ± 14). Higher Pirani score at presentation correlates with lower functional scores (*p* = 0.001). The functional outcome score of children with bilateral club foot was 129.96, compared to a score of 133.7 in children with unilateral involvement, but this was statistically insignificant (*p* = 0.066). There was a correlation between functional score and repeated surgery. Lower scores had more surgery (Pearson correlation −0.312, *p* < 0.001).

The number of casts correlated to the functional assessment. A higher number of casts applied towards correction had lower functional scores (Pearson correlation −0.194, *p* = 0.003).

Child development was assessed by recording the attainment of walking age (Table 2). The average walking age in our group was 15 months (range 10–22 months). One hundred and sixty-four children (88 %) started walking before 18 months of age, while 22 children (12 %) started independent walking between 19 and 22 months of age. There was no correlation between bilateral involvement and delay in walking age.

Parents’ concerns that prolonged use of the braces will interfere with child milestone achievements is not supported by our findings. Parents’ fears of the effect of bracing on a child’s walking age need to be addressed in order to reach better cooperation with the prolonged use of FAB.

## Discussion

FAB use was found to be the most influential parameter in preventing relapse in corrected club foot treated by the Ponseti method [9, 10]. This finding led to the new recommendation of prolonged use of FAB from the initial 2 years to as high an average as 4 years [10]. In this study, we found that, by using the brace for 33 months adopting our weaning protocol, we minimize the risk for relapse. We believe that the rate of surgery in our group could be even lower, since we were not fully aware of the importance of the duration of brace use in the first years of our Ponseti clinic, and followed the 2-year recommendation. Our recommendation was changed later on to a prolonged period of time and we stopped using the brace after 48 months.

In our opinion, the brace weaning protocol should take into account the significance of the delayed ossification of the tarsal bones which was repeatedly shown in many studies [11–19]. The mechanism and pattern of ossification development in the tarsal bone comes from the center of each bone to assume the shape of the cartilaginous anlage of the bone, whereas the latter has already been modeled by extrinsic forces and by biological endochondral ossification programming. It was shown that the critical period includes the first 3 years of the child’s life [11]. In our opinion, the process of ossification of the tarsal bone holds the clue to maintaining the shape of the foot once full correction is achieved by the Ponseti method. We strongly believe that the first stage is correction of the deformity and the second stage is a period of waiting for the foot elements to be ossified in their new proper shape. Paying attention to the individual time schedule of the ossification in club foot is of prime importance to reduce recurrence [11]. There are many studies [14, 15] that report on the delayed ossification process in tarsal bones in club foot. For example, it was shown that club foot talus has a smaller size and the ossification center of the talus is absolutely and relatively smaller. The endochondral ossification sequence in the talus as well as in the calcaneus in the club foot was shown to be disturbed [15, 16]. In club feet, the onset of ossification is significantly earlier in girls than in boys [14]. Ponseti himself reported on this issue in his radiographic study of skeletal deformities in treated club feet [13]. He had studied 32 patients with unilateral club foot followed up between 13 and 30 years. It was shown that many club feet had small talar heads, and undersized or misshapen facets of the subtalar joints. By using the three-dimensional magnetic resonance imaging (3D MRI) technique, it was demonstrated that the total volume of tarsal bone is smaller by about 20 % than in normal feet. The ossification center of the talus has a reduced volume of about 40 % and the calcaneus of 20 %. The length of the talus in club feet is 8.2 % shorter than in normal feet, while the calcaneus is shorter [16–18]. It is possible to predict the shape of the tarsal bone from the shape of the ossified nuclei. MRI multiplanar reconstruction was used to evaluate the anatomy of club feet, especially the talonavicular articulation. An abnormal relationship was demonstrated [20]. In their MRI study, Pirani et al. [21] demonstrate what happens between changes of casts. They were able to show the correction of wedge-shaped talar head (cartilage molding), medial talar neck inclination, as well as the medial navicular displacement. They showed correction of the wedge-shaped distal calcaneal articular surface and the medially displaced cuboid. The inverted calcaneus reverts to a normal position. Once the tarsal bones have enough ossification volume and less soft cartilage, they can resist the deforming forces of the pathological contracting soft tissues. This will maintain the corrected shape of the foot.

In order to achieve this longer time span with the brace use, we developed a model of physiotherapy-led Ponseti club foot clinic. The physiotherapist is the center of the parents’ support group. The physiotherapist provides an intimate interpersonal touch and stronger bond with the family. Successful communication provides support, encouraging higher compliance to the treatment proposed. Parents have easier accessibility through open-to-all phone calls or visits with the physiotherapist. This model provides accurate assessment of the response to treatment and close follow-up, and enables early detection of non-compliance or recurrence that deserves special attention.

The second factor to increase the rate of use of braces is a fully corrected foot before applying the brace. A fully corrected foot will lower complications and difficulties with adjustment to the brace, lower the frustration of the child, and improve compliance. This is the reason for the high rate of Achilles tenotomy in our group. We believe that this is a mandatory part of correcting the foot and only in a very few cases did we choose not to perform Achilles tenotomy. Only in cases where the heel pad was not empty and there was more than 20° of dorsiflexion did we choose not to perform Achilles tenotomy. A high rate of Achilles tenotomy and prolonged use of FAB after correction can lower the rate of recurrence in club foot.

Attainment of walking of children with idiopathic club feet following the Ponseti treatment in infancy was one of our major concerns, as our FAB treatment and weaning protocols were much longer than the protocol proposed by others. We have shown that, in our group of patients, the average walking age is 15 months and 88 % of our children start independent walking before 18 months of age. The remaining children started walking no later than 22 months of age. Several studies show evidence that idiopathic club foot babies were likely to walk a little bit later than typically expected, although walking is likely to occur in the majority of cases by 15–18 months. Garcia et al. [22] and Sala et al. [23] found that independent ambulation was achieved up to 2.2 months later in club foot-treated children and 90 % of the children were walking by 17.7 months. They concluded that the only minimal gross motor delay may occur in the Ponseti-treated children. In a recent study, Zionts et al. [24] showed equivalent results. The mean age at which children began to walk independently was 14.5 (range 10–22 months). By 18 months, 90 % of the patients were independently walking. Of interest is the finding that children with moderate deformity began walking earlier than children with very severe deformity. It is of utmost importance to look into the FAB protocol in each of the studies. Both Sala et al. [23] and Zionts et al. [24] used the FAB full time for 3 months and then during nap time/night time, while Garcia et al. [22] specified that the FAB was worn full time for several months, progressing to sleep time only.

It is clear that the average and range of walking age of children with idiopathic club feet treated with the Ponseti method was the same in our children as in the published studies. This may question the influence of the length FAB of use during the first year of life on the initiation of independent ambulation. Parents’ concern as to the inhibitory effect the FAB might have on the development of their child are not supported by our experience and the above-cited studies, and they should be addressed during the clinic visits, where parents should be provided periodic assessment and reassurance.

When compared with other published studies with the same mean follow-up, our results are similar or slightly better in respect of the need for additional surgery and functional outcomes. In addition, our study had a larger group of patients. Bor et al. [25] treated 74 children (117 feet). After a mean of 6.3 years of follow-up, 24/74 (32 %) patients underwent additional surgical procedures other than tenotomy, compared to 12.9 % in our study. The authors found that the Ponseti brace protocol is essential in order to decrease the rate of additional surgery. Goldstein et al. [26] treated 86 patients (134 feet), with a minimum follow-up of 3 years. Forty-three out of 134 feet (32 %) underwent additional surgery other than tenotomy. The authors also found that compliance with the FAB was the most important factor to avoid additional surgery. Radler et al. [27] treated 113 children (182 feet) with a mean follow-up of 5.2 years. TATT was performed in 24/182 feet (13 %) and open release surgery was performed in the other 9/182 (5 %) feet. The open-joint surgeries were performed in patients belonging to their initial series, representing their learning curve with the Ponseti method.

It is clear that the weak points of our study are it being retrospective and that a prospective controlled study should be designed in order to validate and substantiate our findings. Another limitation is that 43 patients were still in FAB treatment at the time of follow-up. To combat this as a potential confounder, these patients were not included in the analysis that showed a significant correlation between the duration of FAB treatment and relapse requiring surgery. The timing of brace use was calculated according to interviews with parents in 1–3-month intervals.
